# Isotretinoin for rosacea: A systematic review

**DOI:** 10.1016/j.jdin.2024.04.009

**Published:** 2024-05-06

**Authors:** Sapana Desai, Adam Friedman

**Affiliations:** George Washington University Medical Faculty Associates, Department of Dermatology, George Washington University School of Medicine and Health Sciences, Washington, DC

**Keywords:** general dermatology, inflammatory dermatosis, isotretinoin, rosacea, sebocytes, treatment

**Figure:**
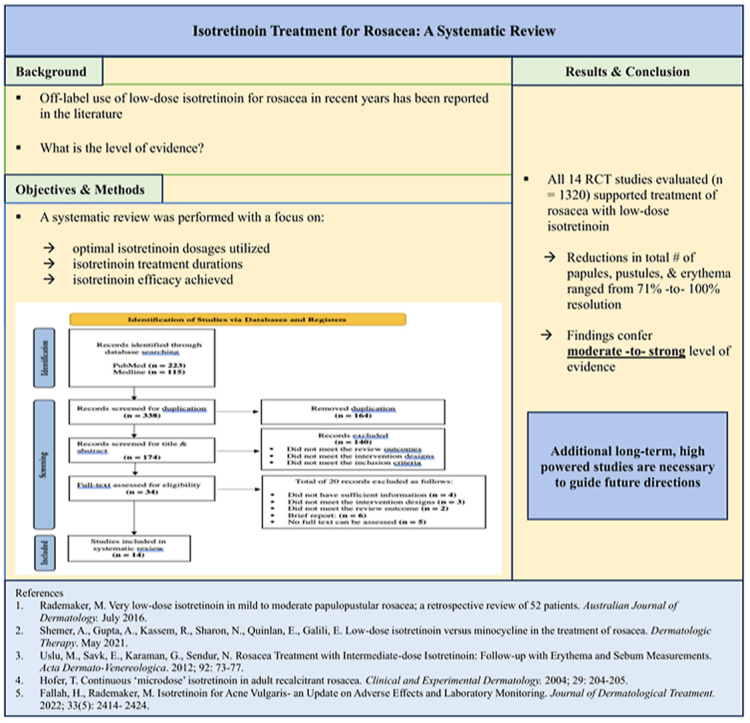

*To the Editor:* Rosacea is a chronic inflammatory dermatosis affecting 10% of the population worldwide. Although global guidelines recommend using oral antibiotics combined with topical anti-inflammatory agents as first-line management for moderate-to-severe disease, recalcitrance is common, requiring off-label therapeutic approaches such as low-dose isotretinoin (LDI). Herein, a systematic review was performed to assess the current level of evidence supporting LDI for rosacea, with focuses on dosages utilized, treatment duration, and efficacy achieved.

Grading of Recommendations, Assessment, Development, and Evaluation (GRADE) assessments was performed using PubMed and MEDLINE, with search terms including “Rosacea AND Isotretinoin.” Study selections were exclusive to randomized clinical trials, retrospective studies, case reports, and case series.

Of 34 studies identified, 14 studies (*n* = 1320; mean age, 36 years [range 18-66 years]; 61% females; Table I) evaluated use of LDI (0.1-0.5 mg/kg/d or fixed doses of 10-20 mg/d) for moderate-to-severe and recalcitrant rosacea for an average treatment duration of 16-weeks or longer. Commonly affected sites among all patients included the cheeks, nose, and forehead. Reductions in total number of papules, pustules, and erythema ranged from 71% to 83% to complete resolution. Increased tolerability was observed at 10 mg/d (*n* = 556), but disease remission periods varied, influenced by use of additional therapies.

**Table I:** Summary of grade of evidence for low-dose isotretinoin in the treatment of rosacea

Citation	Study type	Objectives	Total number of patients	Treatment protocol	Outcomes	Grade of evidence
Ertl G, Levine N, Kligman A. A comparison of the efficacy of topical tretinoin and low-dose oral isotretinoin in rosacea. *Journal of the American Medical Association*. 1994;130(3):319-324.	Randomized, double-blind trial	Evaluate efficacy of topical tretinoin and low-dose ISO in severe and recalcitrant rosacea	*n* *=* 22	Compared low-dose ISO (10 mg/d × 16 wk) vs tretinoin 0.025% cream every day × 32 wk vs combination of low-dose ISO × 16 wk + 0.025% cream × 32 wk	Low-dose ISO worked significantly better than tretinoin 0.025% topical cream and showed rapid rosacea improvement with therapeutic benefits of decreased number of papules/pustules/erythema	2
Gajardo J. Severe rosacea treated with oral isotretinoin. *Revista Medica de Chile.* 1994;122(2):177-179.	Randomized clinical trial	Evaluate efficacy of low-dose ISO in severe rosacea	*n* *=* 6	Compared low-dose ISO (10 mg/d × 3-to-6 mo) vs low-dose ISO (20 mg/d × 3-to-6 mo) Patients were followed up for 2 years	Low-dose ISO at both doses showed significant rosacea improvement at 16 wk with therapeutic benefits of decreased number of papules/pustules/erythema; those on 10 mg/d experienced fewer adverse effects (eg, keratitis) After treatment, patients remained asymptomatic for a mean of 15 mo	2
Gandola M. Therapy of severe acne and acne rosacea with Oral 13-cis-retinoic acid (isotretinoin). *Acta Vitaminol Enzymol.* 1984;6(4):325-337.	Randomized clinical trial	Evaluate efficacy of ISO in severe nodulocystic acne and/or acne conglobota with overlapping components of severe and recalcitrant rosacea	*n* *=* 40	Patients received ISO 40 mg/d (0.66 mg/kg/d) that was later reduced to 2.5 mg/d (0.33 mg/kg/d) for an average of 24 wk (range, 12-40 wk depending on severity)	31 patients (77.5%) experienced complete resolution and ultimate healing; remaining 9 patients demonstrated marked amelioration with decreased number of papules/pustules/erythema 66% of cases experienced increases in total serum cholesterol	2
Golnick H, Szabo E, Meyer K, et al. Systemic isotretinoin in the treatment of rosacea-doxycycline-and placebo-controlled, randomized clinical study*. J Dtsch Dermatol Ges*. 2010;8(7):505-515.	Double-blind, double-dummy, randomized, placebo-controlled 5-arm multicenter combined prospective phase II/III study	Evaluate efficacy of low-dose ISO in moderate-to-severe rosacea	*n* *=* 753	Compared 1 of 3 dosages of low-dose ISO (0.1 mg/kg, 0.3 mg/kg, or 0.5 mg/kg) vs (doxycycline 100 mg every day × 14 d and then 50 mg every day) vs (placebo) × 12 wk	Low-dose ISO 0.3 mg/kg proved to be the most effective dose; showed significant superiority when compared with doxycycline with reduction of 90% of papules/pustules/ erythema/edema vs 83% 24% of cases experienced complete remission vs 14%, and 86% of cases experienced marked improved of seborrhea vs 57%	1
Hofer T. Continuous ‘microdose’ isotretinoin in adult recalcitrant rosacea. *Clin Exp Dermatol.* 2004;29(2):204-205.	Retrospective study^3^	Evaluate efficacy of continuous microdose of ISO in severe and recalcitrant rosacea	*n* *=* 12	Patients received low-dose ISO (10 mg/d or 20 mg/d) × 4-6 mo, followed by ISO microdosing at 0.03-0.17 mg/kg/d	Remission of rosacea was achieved devoid of relapsing episodes; it was safe, and all patients tolerated it without experiencing any adverse effects	2
Hoting E, Paul E, Plewig G. Treatment of rosacea with isotretinoin. *International Journal of Dermatology.* 1986;25(10):660-663.	Open noncomparative multicenter, randomized clinical trial	Evaluate efficacy of low-dose ISO in severe and recalcitrant rosacea	*n* *=* 92	Patients received low-dose ISO 0.5 mg/kg/d × 20 wk and were monitored at weeks 2, 4, 8, 16, and 20	Low-dose ISO was strongly effective in achieving complete resolution in ∼74% of patients, and all patients experienced therapeutic benefits of decreased number of papules/pustules/erythema/telangiectasia/disappearance of sebaceous hyperplasia/improved seborrhea	2
Kwon H, Jung J, Lee W, Bae Y, Park G. Combined treatment of recalcitrant papulopustular rosacea involving pulsed dye laser and fractional microneedling radiofrequency with low-dose isotretinoin. *Journal of Cosmetic Dermatology*. 2020;19(1):105-111.	Retrospective study	Evaluate efficacy of low-dose ISO in severe and recalcitrant rosacea	*n* *=* 25	Patients received low-dose ISO (10 mg/d) × 8 wk and simultaneously received 3 full-face treatments of pulsed dye laser and fractional microneedling radiofrequency at 4-wk intervals	This combination regimen demonstrated satisfactory efficacy; at 16 wk, therapeutic benefits of decreased number of papules and pustules by 71% were observed and erythema index decreased by 54% when compared with baseline	2
McFalda W, Roebuck H. Rational management of papulopustular rosacea with concomitant facial seb. dermatitis: a case report. *J Clin Aesthet Dermatol*. 2011;4(1):40-42.	Case report	Evaluate efficacy of low-dose ISO in moderate rosacea with concomitant facial seborrheic dermatitis	*n* *=* 1	Patient received low-dose ISO (10 mg/d) × 16 wk	Low-dose ISO showed significant rosacea improvement at 16 wk with therapeutic benefits of decreased number of papules/pustules/erythema, as well as decreased seborrhea	6
Nikolowski J, Plewig G. Oral treatment of rosacea with 13-cis-retinoic acid. *Hautarzt.* 1981;32(11):575-584.	Randomized Clinical Trial	Evaluate efficacy of low-dose ISO in severe and recalcitrant rosacea	*n =* 13	Compared low-dose ISO 0.05 mg/kg vs 0.5 mg/kg vs 1 mg/kg × 12-28 wk	Low-dose ISO showed significant rosacea improvement at 16 wk with therapeutic benefits of decreased number of papules/pustules/erythema/telangtasias Lesions regressed by 50% within 2 wk and by >95% within 8 wk	2
Rademaker M. Very low-dose isotretinoin in mild to moderate papulopustular rosacea; a retrospective review of 52 patients. *Australas J Dermatol*. 2018;59(1):26-30.	Randomized clinical trial^1^	Evaluate efficacy of low-dose ISO in mild-to-moderate rosacea	*n* *=* 52	Patients received low-dose ISO (20 mg/d) which was then reduced to 10 mg/d in 67% of patients and increased to 30-40 mg/d in 15% of patients	Low-dose ISO 10-20 mg/d showed rosacea clearance in 91% of all cases; 31% of all patients completed a second course of ISO after a minimum of being off the medication for 6 mo	2
Sbidian E, Vicaut E, Chidiack H, Anselin E, Cribier B, Dreno B, Chosidow O. A randomized controlled trial of oral low dose isotretinoin for difficult to treat papulopustular rosacea. *J Invest Dermatol.* 2016;136(6):1124-1129.	Multicenter, double-blind, randomized-placebo-controlled trial	Evaluate efficacy of low-dose ISO in severe and recalcitrant rosacea	*n* *=* 156	108 patients received low-dose ISO 0.25 mg/kg/d × 16 wk and 48 patients received placebo × 16 wk	57.4% of the 108 patients on low-dose ISO experienced ∼90% reduction in papules and pustules when compared with baseline; among the ISO-treated responders, remission was complete for 34 patients and partial for 17 patients 58.3% of patients receiving placebo relapsed with a median of 15 wk to reoccurrence	2
Schell H, Vogt H, Hennes A. Treatment of rosacea with isotretinoin. Results of a multicenter trial follow-up. *Z Hautkr.*1987;62(15):1123-1124.	Multicenter, randomized clinical trial follow-up	Evaluate efficacy of low-dose ISO in severe and recalcitrant rosacea	*n* *=* 47	All patients formerly treated with low-dose ISO (0.5 mg/kg/d) × 12-20 wk were followed up at 57 wk after treatment	40 patients formerly treated with low-dose ISO for rosacea remained in remission even after 57 wk after treatment and continued to experience therapeutic benefits of resolution of inflammatory papules/pustules/erythema	2
Shemer A, Gupta AK, Kassem R, Sharon N, Quinlan EM, Galili E. Low-dose isotretinoin versus minocycline in the treatment of rosacea. *Dermatol Ther*. 2021;34(4):e14986.	Retrospective comparative study^2^	Evaluate efficacy of low-dose ISO in mild-to-moderate and severe recalcitrant rosacea	*n* *=* 76	Patients with mild-to-moderate rosacea (*n* = 28) received ISO 20 mg/wk; patients with severe recalcitrant rosacea (*n* = 24) received ISO 40 mg/wk; and 24 patients received minocycline 100 mg/d All treatment courses lasted 4-7 months	All treated rosacea cases showed appreciable improvement Patients treated with low-dose ISO 20 mg/wk achieved a significantly higher GIS score when compared with minocycline 100 mg/d Patients treated with ISO 40 mg/wk demonstrated complete response rates of 62.5%	3
Uslu M, Savk E, Karaman G, Sendur N. Rosacea treatment with intermediate-dose isotretinoin: follow-up with erythema and sebum measurements. *Acta Derm Venereol*. 2012;92(1):73-77.	Randomized clinical trial^4^	Evaluate efficacy of low-dose ISO in recalcitrant rosacea	*n* *=* 25	Patients received low-dose ISO 20 mg/d × 16 wk that was then tapered for the subsequent 6 mo	Low-dose ISO showed significant rosacea improvement beginning at 4 wk with therapeutic benefits of decreased number of papules/pustules/erythema/seborrhea Within a mean follow-up time of 11 mo, 45% of patients underwent relapses; 55% of patients continued to remain in remission	2

*GIS*, Global improvement scale; *ISO*, isotretinoin.

One retrospective study (*n* *=* 52) assessing open-label LDI treatment for 57-weeks (initial dose of 20 mg/d, with dose adjustments made accordingly with responses) in patients with mild-to-moderate rosacea yielded significant improvement. Moreover, 91% of patients experienced complete clearance of papulopustules and/or 75% to 99% reduction of papulopustules.^1^ Although a patient’s treatment was individualized by altering LDI dosages to specifically control their symptoms and any concomitant diseases (acne and/or seborrheic dermatitis), 71% of patients reduced their isotretinoin dose to 10 mg thrice weekly within 3 months of starting therapy, which they continued for 1 year.^1^

Another comparative study (*n* *=* 76) evaluated efficacy of once weekly administration of LDI (20 mg/wk) versus minocycline (100 mg/d) for mild-to-moderate rosacea and LDI (40 mg/wk) for severe rosacea for a 7-month duration.^2^ Despite appreciable improvement in all treated cases, 20 mg/wk isotretinoin achieved a statistically significant higher mean global improvement scale score when compared with 100 mg/d minocycline (global improvement scale, 2.8 vs 2.3, respectively; *P* < .05) but minimal differences between complete response rates (10.7% vs. 8.3%, respectively; *P* = .77). Patients treated for severe rosacea demonstrated a complete response rate of 62.5% and a mean global improvement scale of 3.7.^2^ An additional study (*n* *=* 22) used continuous isotretinoin microdosing (0.03-0.17 mg/kg/d) following isotretinoin 10 to 20 mg/d that also was promising.^3^

All evaluated studies supported treatment of rosacea with isotretinoin. Isotretinoin can reduce sebaceous gland size and activity and even induce apoptosis and downregulate pattern recognition receptor expression implicated in disease pathophysiology conferring anti-inflammatory activity (Fig 1).^2^^,^^4^^,^^5^ Given that the selected studies primarily encompassed randomized clinical trials, each having marginal risk of biases, demonstrating consistency, and generating similar clinical outcomes, these findings confer a moderate-to-strong level of evidence supporting isotretinoin effectiveness to treat recalcitrant rosacea. However, routine LDI implementation is limited by lack of standardized protocols thus warranting additional long-term, high-powered studies to guide future directions and consensus on optimal dosages and treatment intervals needed to maintain disease clearance.

**Fig 1:**
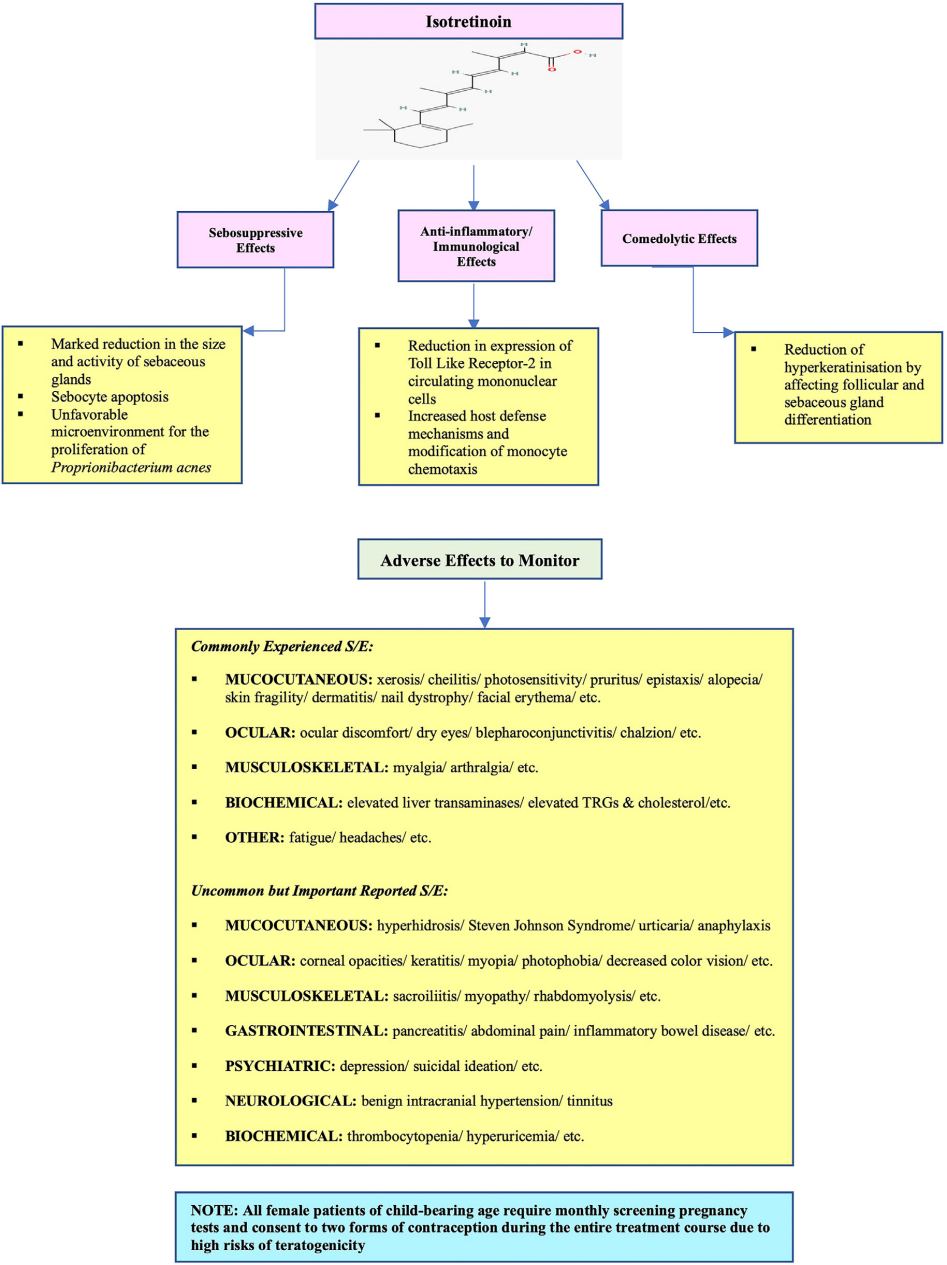
Favorable effects of isotretinoin and its safety.^2^^,^^4^^,^^5^

## Conflicts of interest

None disclosed.
